# Searching standard parameters for volumetric modulated arc therapy (VMAT) of prostate cancer

**DOI:** 10.1186/1748-717X-7-108

**Published:** 2012-07-11

**Authors:** Marius Treutwein, Matthias Hipp, Oliver Koelbl, Barbara Dobler

**Affiliations:** 1 Department of Radiation Oncology, Regensburg University Medical Center, Franz-Josef-Strauss-Allee 11, 93053, Regensburg, Germany

**Keywords:** VMAT, Volumetric modulated arc therapy, Optimization, Prostate, Simultaneous integrated boost

## Abstract

**Background:**

Since December 2009 a new VMAT planning system tool is available in Oncentra® MasterPlan v3.3 (Nucletron B.V.). The purpose of this study was to work out standard parameters for the optimization of prostate cancer.

**Methods:**

For ten patients with localized prostate cancer plans for simultaneous integrated boost were optimized, varying systematically the number of arcs, collimator angle, the maximum delivery time, and the gantry spacing. Homogeneity in clinical target volume, minimum dose in planning target volume, median dose in the organs at risk, maximum dose in the posterior part of the rectum, and number of monitor units were evaluated using student’s test for statistical analysis. Measurements were performed with a 2D-array, taking the delivery time, and compared to the calculation by the gamma method.

**Results:**

Plans with collimator 45° were superior to plans with collimator 0°. Single arc resulted in higher minimum dose in the planning target volume, but also higher dose values to the organs at risk, requiring less monitor units per fraction dose than dual arc. Single arc needs a higher value (per arc) for the maximum delivery time parameter than dual arc, but as only one arc is needed, the measured delivery time was shorter and stayed below 2.5 min versus 3 to 5 min. Balancing plan quality, dosimetric results and calculation time, a gantry spacing of 4° led to optimal results.

**Conclusion:**

A set of parameters has been found which can be used as standard for volumetric modulated arc therapy planning of prostate cancer.

## Background

Volumetric modulated arc therapy (VMAT) is a rather novel form of radiotherapy delivery, varying multi leaf collimator (MLC) shape, dose rate and gantry speed simultaneously during gantry rotation. Otto presented this technique in 2008 [1]. Up to that date intensity modulated radiation therapy (IMRT) had emerged as a standard technique for radiotherapy of the prostate [2-6]. Shorter treatment times, which are desirable with regard to intrafractional organ motion, are possible with VMAT [1,7-10]. A few studies have been published comparing IMRT and VMAT for prostate radiotherapy [11-15]. All of them used data of Varian accelerators with its implementation of VMAT called RapidArc® (Varian Medical Systems, Inc., USA) and either prototypes of planning or optimizing systems or Varian’s Eclipse™. Otto stated that the ability to generate complex dose distributions is highly dependent on the optimization algorithm and capabilities of the delivery system used for treatment [16,17]. A new system combination of two other manufacturers became clinically available for VMAT in December 2009: a VMAT treatment planning tool implemented in Oncentra® MasterPlan v3.3 (Nucletron B.V., Veenendal, The Netherlands), and the VMAT implementation on a Synergy®S linac (Elekta Ltd, Crawley, UK). First results achieved with this system combination have been published for a limited number of patients [18,19]. Planning studies with statistical significance are, however, not yet available.

The aim of our study was to assess the potential of this new system combination for VMAT of prostate cancer. The treatment planning was performed for a simultaneous integrated boost treatment for 10 patients. A systematic variation of the user defineable parameters was performed to identify the influence of the different parameters on plan quality, treatment time and monitor units (MU). A detailed evaluation of 360 treatment plans allowed the deduction of the optimal parameter set for VMAT optimization with statistical significance.

## Methods

### Patients and regions of interest (ROI)

Ten consecutive patients with a mean age of 71 years, which had been treated with primary external-beam radiotherapy for localized prostate cancer, were included in the retrospective planning study. Written informed consent was obtained from the patients for publication of this report and any accompanying images. All ten patients were immobilized in a vacuum mattress (Blue-BAG™ BodyFIX®, Medical Intelligence, Schwabmünchen, Germany) in supine position according to Boehmer et al. [20] and had three-dimensional treatment planning with a CT slice thickness of 5 mm. In the treatment-planning system Oncentra® MasterPlan, v3.3 (Nucletron B.V., Veenendaal, The Netherlands) the gross target volume (GTV: prostate gland and seminal vesicles), the clinical target volume (CTV, 5-mm three-dimensional margin added to the GTV excluding the rectal volume), the planning target volume (PTV, 10-mm three -dimensional margin added to the GTV without respect to the rectum), and normal tissues were delineated in each slice. The delineation of the volumes of interest followed the description of Bos et al. [21], who also showed that a little overlap of PTV and rectum results from the lateral expansion of the PTV. The rectal volume (according to Guckenberger et al. [22]) and urinary bladder as well as the femoral heads were delineated as organs at risk (OAR). Additional regions were defined to improve the plan quality: the PTV plus a margin of 5 mm (PTVm) was first subtracted from the patient outline (OL) for a non target volume (OL-PTVm) to avoid hot spots in the normal tissue; It was second subtracted from the rectum volume resulting in the posterior part of the rectum (R-PTVm) to achieve reduced dose in this region as similarly described in [12]. The last structure constructed was PTV minus CTV (PTV-CTV) to model the dose gradient from PTV to CTV.

### Linear accelerator

For planning and measurements the data of a Synergy®S linear accelerator (Elekta Ltd, Crawley, United Kingdom) with 6MV photons were used, equipped with a BeamModulator™ head, an iViewGT™ electronic portal imaging device, and an on-board cone-beam CT XVI. The multileaf collimator has 40 leaf pairs, each of nominal width of 4 mm projected to isocentre, diaphragms limit the maximum field size of 21 cm × 16 cm. There are no moveable jaws. Leaf interdigitation is allowed without limitations, which means that each leaf can travel across the whole field size, independent on the position of neighbouring leaves. Closed leaf pairs are automatically shifted below the fixed diaphragm to minimize transmission between the leaf ends of opposed leaves.

The following linac specific parameters for VMAT delivery have to be fed into the planning system as described similar for Eclipse™ and RapidArc® [23]:

Minimum and maximum number of MU per degree of gantry rotation 0.10 MU/° and 20.0 MU/° respectively, minimum MU per cm leaf travel 0.30 MU/cm, maximum gantry speed 6.00°/s. Maximum leaf speed is 2.4 cm/s, the dynamic minimum leaf gap 0.2 cm, and the static minimum leaf gap 0.0 cm [24]. The maximum nominal dose rate is 500 MU/min. Seven fixed dose rate levels are available, each half the dose rate of the next higher level, continuous variation is not possible. Actual dose rates may differ from nominal dose rates by ±25%. For VMAT delivery the console software Precise Desktop® 7 determines automatically the fastest combination of dose rate, gantry speed and leaf speed [19].

### Treatment planning system (TPS)

The treatment planning was performed with Oncentra MasterPlan® v3.3 SP1, released clinically in December 2009, on a 64 bit Windows system. From the above mentioned data the two lowest dose rate levels had to be omitted, as the TPS allows only five different levels [19]. In the Beam Modeling module the user appoints only the treatment unit, the number of beams, the energy and the collimator angle. Although the linac could vary the collimator angle during VMAT delivery this feature is not implemented in the current TPS software version. Switching to the Optimizer module the user has further parameters to determine: gantry start angle, rotation direction, arc length, gantry angle spacing between subsequent control points (2°, 3°, 4°, or 6°), maximum delivery time (MDT), number of arcs, and leaf motion constraint in cm/°, which was set to 0.5 cm/°. The optimizer called RayArc has been developed by RaySearch and is described in [25] and in a White Paper [26]. RayArc is designed to create deliverable plans for Elekta and Varian machines. It exists in two versions, as SmartArc module integrated in Pinnacle³ Version 9 (Philips Healthcare, The Netherlands) and the mentioned Oncentra version.

### Planning

The plans were set up with simultaneous integrated boost (SIB) in 33 fractions aiming for 59.4 Gy minimum dose to the PTV and 71.0 Gy minimum dose and 74.2 Gy maximum dose to the CTV, which was used as the boost treatment volume. As in [13,18] and also in [25] (with little modifications) the same set of dose volume objectives (DVO) was used (Table 1) for all VMAT and IMRT plans, based on experiences with the same optimization module for IMRT [27]. The DVO are set as planning goals to be met if possible. As Dobler et al. [28] stated it might be possible to achieve better plan quality using DVO specifically designed for the algorithm and delivery method. However, a direct comparison of results would be impossible.

**Table 1:** Dose volume objectives used for the optimization

** *ROI* **	** *Type* **	** *Dose Level (Gy)* **	** *Volume (%)* **	** *Weight* **
PTV-CTV	Minimum Dose	59.4	100	3,000
PTV-CTV	Maximum Dose	71.0	0	3,000
CTV	Minimum Dose	71.0	100	3,000
CTV	Maximum Dose	74.2	0	3,000
Urinary Bladder	Maximum Dose Volume	50	50	1,000
Rectum	Maximum Dose	74.2	0	1,000
Rectum	Maximum Dose Volume	70	20	1,000
Rectum	Maximum Dose Volume	50	60	1,000
R-PTVm	Maximum Dose	50	0	1,000
Left and right femoral head	Maximum Dose Volume	50	50	300
OL-PTVm	Maximum Dose	56	0	3,000
OL-PTVm	Maximum Dose Volume	45	5	3,000

Dose volume objectives and their weights used in the objective function during the optimization process.

The isocentre was positioned in the centre of the CTV. All calculations were performed by a pencil beam algorithm with a calculation grid spacing of 4 mm. Both the slice thickness of 5 mm and the calculation grid spacing of 4 mm were rather coarse regarding a leaf width of 4 mm. The reason is that the main memory of the hardware was too small to handle a better resolution in the optimization process. To verify that there was no misinterpretation by interpolation, for a subset of ten different plans, one for each patient, a forward calculation was done with a finer resolution. An artificial CT with a slice thickness of 2.5 mm was generated by means of the TPS. The calculation grid spacing was set to 2 mm. All plans were forward calculated without new optimization and without any change. There was either no change, or the values shifted slightly for all plans systematically to the same direction, keeping the general conclusion unchanged.

Altogether 360 plans were calculated, 36 per patient. The following parameters were varied systematically for each patient:

#### Single versus dual arc

Besides the option to deliver the treatment in a single arc (SA), it is also possible to choose two or multiple arcs. They can be used as two “independent” arcs or as dual arc (DA). As Dobler et al. [19] and Eriksson et al. [26] have described, for targets divided by an OAR in a left and a right side (as given by prostate and rectum) in the DA mode one arc will focus to the left and one to the right side, reducing the leaf openings over the OAR and thereby the dose delivered to it. Two independent arcs on the other hand would both produce similar segments around the arc. Therefore SA and DA were the chosen alternatives in our study both rotating from 182° to 178° respectively bidirectional.

#### Collimator angle

Otto stated [1] and confirmed later [16] that 45° collimator angle has been found to be preferable in most cases. Bortfeld [29] comments on this question that it is not completely clear where the advantage of a 45° collimator comes from. In [30] Bortfeld and Webb have done their calculations with a collimator angle of 0° confining to a 2D model. Otto [16] extended the analysis to the capabilities of RapidArc® including a collimator angle of 45°. Since this still seems to be analyzed only theoretically, all plan optimizations were performed for collimator 45° and 0°.

#### Maximum delivery time

The MDT is an optimization parameter allowing the user to make a delivery time restriction. It is only an estimate for the real delivery time, as gantry speed and dose rate values are determined by the console software using the control point information [25]. Due to limitations in gantry speed of 6.00°/s, a rotation around 356° requires at least 59.3 s. To allow some more time for modulation a minimum value per arc of 80 s was chosen, slightly more than recommended by Eriksson et al. [26] (75 s). As a medium value we selected 110 s and as maximum value 150 s.

#### Gantry spacing

The gantry spacing (GS) between two subsequent control points is reduced during the optimization process. Starting with increments of 24 degrees additional control points are added to achieve the final GS selected by the user [31]. As Eriksson et al. [26] recommend to start with a spacing of 4 degrees, we decided to increase and decrease to the extreme values possible in the TPS and used three different GS: 2°, 4°, and 6°. These are also the values which Feygelman et al. [32] used for their SmartArc evaluation and allow comparison to their results.

#### IMRT

For comparison one IMRT plan was calculated for each patient applying direct step and shoot optimization also using the DVO of Table 1. The collimator was in standard position of 0°. The planning parameters followed Treutwein et al. [27]: seven equispaced beams, starting at a gantry angle of 0°, followed by 51°, 103°, 154°, 206°, 257° and 309°; minimum field size of 4 cm², at least two open leaf pairs, maximum number of 60 segments and minimum 4 MU per segment.

### Plan evaluation and statistics

Besides the median values in the OAR the following parameters were evaluated: the total number of MU, the homogeneity H and D_5_ and D_95_ in the CTV with H defined as H = (D_5_–D_95_)/D_Average_[33,34]. D_5_ and D_95_ are defined as the dose to 5% and 95% of the CTV, respectively. Furthermore the maximum dose to the posterior part of the rectum and the minimum dose in the PTV were analyzed as endpoints of the optimization. The results of all plans were grouped according to the modified parameters and analyzed statistically using SPSS PASW Statistics programme version 18.0.0. Groups were compared applying student’s test (*t*-test) for paired samples. As level of significance α = 5% (two-sided) was chosen. To obtain a good statistical significance as few groups of plans as possible were established for each parameter.

Moreover all plans meeting the endpoints mentioned above were selected and the incidence in the different groups was determined.

### Plan verification

Five groups of VMAT plans and the IMRT group containing ten plans for the different patients each were measured with a MatriXX Evolution® 2D-array with gantry angle sensor (IBA Dosimetry, Schwarzenbruck, Germany) [35,36] positioned in the horizontal isocentre plane. The gantry angle sensor was attached to the gantry and connected to the array. Every 200 ms a dose matrix and the corresponding gantry angle were acquired automatically. The matrices were corrected for angular dependencies, including couch attenuation as described in [35], using a correction factor matrix implemented in the software. Lastly they were summed up to the dose of the complete plan. This was compared to the calculated dose matrices by gamma evaluation [37] with a dose tolerance of 3% of the maximum dose and a distance to agreement of 3 mm. We performed the evaluation for the area with dose values above 10% as recommended in [38]. The percentage of pixels out of range (γ > 1) was analyzed. In addition radiation times were measured from pressing the start button to end of delivery.

## Results

### Plan evaluation

All results are shown in Table 2; significant differences between groups are described in the corresponding sections. On behalf of brevity, differences and corresponding p-values are not mentioned if not statistically significant except of an intermediate range (α = 10%), otherwise they are described as significant (α = 5%) or highly significant (α = 1%).

**Table 2:** Average values of the evaluation grouped by the modified parameters

	** *Plan Group* **	** *CTV H* **	** *PTV D* **_** *min* **_	**R-PTVm *D* **_** *max* **_	** *R D* **_** *50* **_	** *B D* **_** *50* **_	** *MU* **
A.	SA	6.1 ± 1.6	59.0 ± 1.6	50.1 ± 2.4	37.0 ± 3.7	37.8 ± 11.4	553 ± 74
	DA	6.1 ± 1.5	58.5 ± 1.9	49.0 ± 2.3	34.9 ± 3.0	37.0 ± 10.3	708 ± 65
B.	C 0°	6.7 ± 1.7	58.5 ± 1.9	50.0 ± 2.5	35.8 ± 3.8	37.2 ± 11.1	655 ± 105
	C 45°	5.5 ± 1.1	59.0 ± 1.6	49.2 ± 2.3	36.1 ± 3.3	37.6 ± 10.7	605 ± 97
C.	MDT 80 s SA	6.5 ± 2.0	58.7 ± 1.7	50.7 ± 2.6	39.1 ± 4.0	39.1 ± 11.9	541 ± 85
	MDT 110 s SA	5.9 ± 1.5	59.2 ± 1.3	50.0 ± 2.2	36.6 ± 3.2	37.6 ± 11.4	554 ± 67
	MDT 150 s SA	5.9 ± 1.0	59.0 ± 1.6	49.6 ± 2.2	35.3 ± 2.8	36.7 ± 11.0	564 ± 67
	MDT 80 s DA	6.0 ± 1.5	58.8 ± 1.6	48.9 ± 2.3	34.9 ± 3.9	37.2 ± 10.3	696 ± 63
	MDT 110 s DA	6.1 ± 1.5	58.5 ± 1.9	49.3 ± 2.3	34.8 ± 3.0	36.9 ± 10.5	710 ± 65
	MDT 150 s DA	6.1 ± 1.6	58.2 ± 2.1	48.9 ± 2.2	35.1 ± 3.1	36.9 ± 10.3	717 ± 68
D.	GS 2°	6.5 ± 1.9	58.8 ± 1.8	49.8 ± 2.7	36.4 ± 4.1	38.1 ± 11.1	650 ± 107
	GS 4°	5.9 ± 1.3	59.0 ± 1.6	49.5 ± 2.2	35.7 ± 3.4	37.2 ± 10.9	626 ± 104
	GS 6°	5.9 ± 1.3	58.4 ± 1.8	49.4 ± 2.2	35.8 ± 3.0	36.8 ± 10.8	614 ± 99
E.	IMRT	6.0 ± 0.7	58.4 ± 1.7	51.9 ± 1.4	44.3 ± 2.6	41.2 ± 13.3	498 ± 25

The first column contains the identifying capital letter for the corresponding paragraph in the results section. Average values and empirical standard deviation are given for the homogeneity H to the CTV (CTV H), the minimum dose to the PTV (*PTV D*_*min*_*)*, the maximum dose to the posterior rectum (R-PTVm *D*_*max*_*)*, the median dose to the rectum (*R D*_*50*_*)* and urinary bladder (*B D*_*50*_), and the MU. Dose values are given in Gy, H in%.

The median dose to the femoral heads in a range from 24.5 Gy to 33.4 Gy as average for the subgroups of ten patient plans stayed far below the DVO of 50 Gy. According to Kjær-Kristoffersen et al. [13] this parameter was disregarded furthermore.

#### Single versus dual arc

All plans with SA optimization were gathered in one group and compared to the corresponding plans with DA resulting in 180 pairs of plans. Differences between SA and DA were highly significant for the following parameters: SA resulted in higher minimum dose in the PTV requiring less MU per fraction dose than DA. On the other hand the maximum dose to the posterior rectum, the median dose to the complete rectum and the median dose to the urinary bladder were highly significant reduced for DA. Both groups contained 37 plans fulfilling the DVO.

#### Collimator angle

All plans with collimator 0° were combined in one group and compared to the corresponding plans with collimator 45° giving 180 pairs of plans. Plans with collimator 45° had a highly significant improvement in the following parameters: homogeneity to the CTV, minimum dose to the PTV, maximum dose to the posterior rectum, and MU per fraction. A majority of 48 plans with collimator 45° met the DVO, but only 26 plans with collimator 0°.

#### Maximum delivery time

As the chosen value for MDT limits the time *per arc* the plans with SA and with DA had to be evaluated separately. Having three MDT levels we got six groups, containing 60 plans each.

First the SA groups were compared. The dose homogeneity in the CTV for MDT 80 s was significantly worse than for MDT 150 s and MDT 110 s. Also the minimum dose to the PTV was in this group significantly lower than for MDT 110 s. The values for the maximum dose to the posterior rectum differed all significantly. Similar were the results for the median dose to the rectum, all differences being highly significant. The average of the median values for the urinary bladder was significantly higher the lower the MDT is. The MU decreased with the MDT, significant comparing MDT 150 s with the two other groups and not significant in the last comparison with p = 9.9% (MDT 110 s versus MDT 80 s). 16 plans with MTD 150 s complied with the DVO, less in the two other groups (10 respectively 11).

In the DA groups the minimum dose to the PTV reached in average the best value for MDT 80 s, which was significantly better than with MDT 150 s. The MU decreased with the MDT, but with p = 8.6% (MDT 150 s versus MDT 110 s) not significant in one case, highly significant for the other two comparisons. The absolute number of plans meeting the DVO was nearly equal in each group: 12 (MDT 150 s), 13 (MDT 110 s), 12 (MDT 80 s).

#### Gantry spacing

Three groups with a GS of 2°, 4° and 6° were evaluated, containing 120 plans each. The average of the homogeneity to the CTV was highly significant higher for GS 2° and identical for the two other groups. The average values for the minimum dose to the PTV were rather close, revealing the group with GS 6° significantly lower versus GS 2° and highly significant compared to GS 4°. Rather close values were found for the maximum dose to the posterior rectum, two pairings differing significantly, GS 2° versus GS 4° and GS 2° versus GS 6°. Similar were the relations for the median dose to the rectum, giving significant differences in two pairings GS 2° versus GS 4° and GS 2° versus GS 6°. In the urinary bladder the values for the median dose were significantly different for GS 4° versus GS 6° and highly significant for the other two cases. The MU decreased with increasing GS. Here all differences were highly significant. The least plans meeting the DVO were found with GS 6° (21), the incidence in the two other groups was nearly equal: 26 (GS 4°), 27 (GS 2°).

#### IMRT

This group contains only ten plans, because the parameters of VMAT optimization are not applicable. Table 2 lists the results in the last line.

### Graphical evaluation

**Figure 1:**
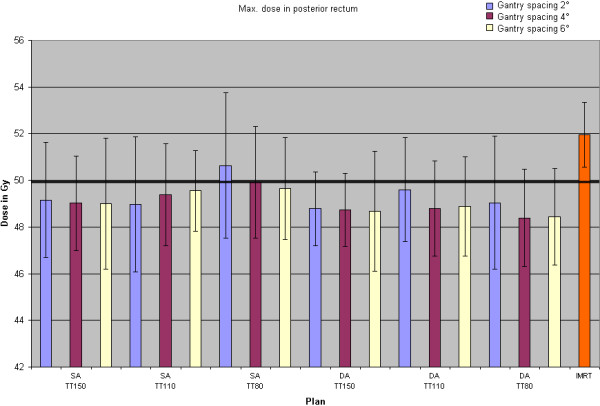
**Maximum dose to the posterior rectum (a.) and minimum dose to the PTV (b.) for different groups of plans with single arc (SA) respectively dual arc (DA) technique, maximum delivery times (MDT) of 150 s, 110 s, and 80 s.** The colour indicates the gantry spacing; the last bar represents the values of the IMRT group. The experimental standard deviation is indicated by error bars. Average values for 10 patients.

**Figure 2:**
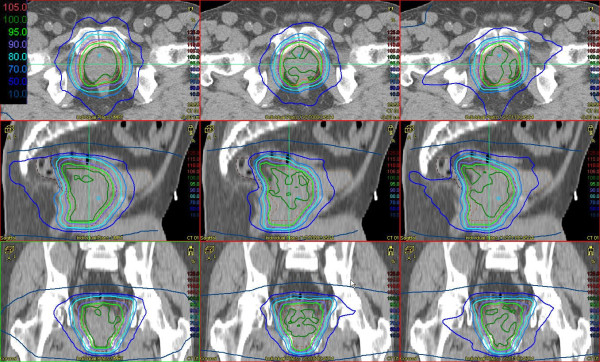
**Dose distributions in three planes (first row: transversal, second row: sagittal, third row: coronal) for one specific patient.** The PTV is delineated in red, the CTV in orange. The IMRT plan is shown in the left column, VMAT [DA, MDT 80 s, GS 4°] in the central column and VMAT [SA, MDT 110 s, GS 4°] in the right column.

In a last step for each parameter combination the plans of the ten different patients were evaluated and the average values for the specified parameters graphically depicted. Our examples (Figure 1) show the bar charts of the endpoints, which did not meet the specified DVO in every case as average over ten plans. For the sake of clarity, the bars of the groups with collimator 0° have been omitted, as it had been shown above that they gave poorer results. The minimum dose in the PTV was only met in two groups, [SA, MDT 110 s, GS 2°] and [SA, MDT 110 s, GS 4°]. Two groups with DA technique remained just under the prescribed value of 59.4 Gy: [DA, MDT 80 s, GS 2°] and [DA, MDT 80 s, GS 4°]. Closest to this value with GS6° was the group [SA, MDT 80 s, GS 6°] reaching 59.3 Gy. Furthermore these five groups fulfilled the DVO for the posterior rectum. Figure 2 shows the dose distributions for one specific IMRT plan, and two VMAT plans ([DA, MDT 80 s, GS 4°] and [SA, MDT 110 s, GS 4°]).

### Plan verification

The four groups resulting as the optimal groups of VMAT from the graphical evaluation, the just mentioned group with GS 6° [SA, MDT 80 s, GS 6°] and the IMRT group were selected for plan verification. Table 3 shows the average values and standard deviations for the delivery times and the percentage of pixels out of range.

**Table 3:** Results of the measurements

** *Plan group* **	** *Delivery time in s* **	** *Pixels out of range in %* **
SA, MDT 110 s, GS 2°	123 ± 6	1.8 ± 1.2
SA, MDT 110 s, GS 4°	124 ± 7	2.5 ± 0.8
DA, MDT 80 s, GS 2°	252 ± 26	1.1 ± 1.6
DA, MDT 80 s, GS 4°	210 ± 8	2.6 ± 1.6
SA, MDT 80 s, GS 6°	86 ± 1	3.5 ± 1.9
IMRT	417 ± 33	2.3 ± 1.1

Average values and standard deviation of the delivery times and percentage of pixels out of range in the gamma evaluation with γ > 1 (3 mm, 3%) for four groups of VMAT plans and for the IMRT group.

## Discussion

The results of the SA and DA comparison do not show a clear advantage of one technique over the other.

Nevertheless the differences are highly significant: a slight benefit for SA at the minimum dose in the PTV which is yet below the specified value in the DVO is in opposition to lower dose values in the rectum for DA. In most cases the minimum dose to the PTV is localized in the posterior region. Keeping the minimum dose here is correlated to a higher dose to the rectum. The DA technique favours the dose reduction by focusing on one half of the PTV for each arc. This is promoted by a higher MDT. A decrease of the objective weight for the posterior part of the rectum and an increase of the weight for the minimum dose in the PTV might improve the DA results. This could be a future step to find an optimal set of DVO.

SA optimization comes out with fewer MU than DA, because DA focuses on one half of the PTV for each arc. Fewer MU for SA treatments are an indicator for shorter treatment times. Additional time is also needed by the record and verify system and the control system of the linear accelerator to prepare the second arc. These circumstances could be confirmed in the plan verification measurements: The treatment times for the selection of SA were all smaller than 2 min 15 s, whereas all DA treatments took more than 3 min 10 s up to nearly 5 min and are therefore above the level of three minutes where considerable organ movements might occur. The studies of Ghilezan [39] and Nederveen [40] have shown an influence of the delivery time on intrafraction organ motion: the longer the fractional treatment lasts the higher is the risk of anatomic deviation. Kupelian [41] argued with this potentially mismatch for daily image guidance and adaptive radiotherapy. In contrast actually there exists no defined standard recommendation for online correction in daily practice when using a VMAT technique for treating prostate cancer. An option to increase the minimum dose to the PTV in two arcs might be using orthogonal collimators for each arc. However, DA is not possible with different collimator values.

Regarding the collimator angle there is a clear outcome that an angle of 45° should be preferred to an angle of 0°. It is first advantageous for the dose distribution; this might be explained by the hypothesis that the leaves of the MLC in parallel opposed beams move in orthogonal directions and therefore these beams are not redundant [29]. Furthermore Otto explains [16] that without collimator rotation only a single leaf pair can be used to modulate the intensity within one CT slice. And second the number of MU is 8% lower with collimator 45° than using a collimator angle of 0° which can be explained by the fact that with collimator 45° it is possible to irradiate the right and left side of the PTV at the same time sparing the rectum and urinary bladder in the centre, which is not possible with collimator 0°.

For SA treatments a MDT of 80 s seems to be too short to achieve an acceptable dose distribution. All evaluated dose parameters were in most cases significantly worse than in the two other groups. Only for the number of MU the best value is achieved. This is not surprising, as there are only two options for the optimizer to reduce the treatment time: higher dose rates and fewer MU. They are closely interconnected during the optimization process [26]. Although a higher value than 80 s for the MDT is necessary, the decision between MDT 110 s and MDT 150 s is not clear without ambiguity: the dose distribution in the CTV and PTV is nearly equivalent, the doses to the OAR are a little bit higher for MDT 110 s, but the MU are lower than for MDT 150 s. A slight advantage for MDT 150 s might be derived from the incidence of plans meeting the endpoints. However, regarding the importance of short treatment times as discussed above and the slight difference in the dose values a MDT 110 s seems preferable, which is affirmed by the graphical evaluation.

The decision in the DA groups is clear: the significantly highest minimum dose to the PTV is achieved with MDT 80 s in two arcs, yielding very similar results for the homogeneity to the CTV and the dose values to the OAR without statistically significant differences and again the lowest number of MU which is statistically significant. Consequently the shortest MDT provides the best results.

It might be supposed that for a given MDT all delivery times shorter are considered by the optimizer. Our measurements indicate that this maximum is exploited. A long MDT results in more arc sections with a dose rate at the lower bound allowing less modulation between the control points. Therefore plans with a longer MDT can be worse than others with a shorter one.

Analyzing the results of the GS comparison we find the best value for the minimum dose to the PTV for GS 4°, here the group with GS 6° has the significantly lowest value. However, in this group we achieve the significantly lowest median dose to the urinary bladder and the lowest number of MU. Nevertheless the advantage of GS 4° versus GS 2° is quite small and might only be traced back to the lower modelling accuracy and might disappear in delivery. Regarding the results of the measurements (Table 3) we find the better accordance for the γ value the smaller the GS. However this is only significant for the DA technique (p = 1.0%), not significant (p = 6.8%) for the SA technique using MDT 110 s, either for GS 6° compared to the other SA measurements. The passing rates are similar as at Feygelman et al. [32] between 95.6% and 100.0% for GS 2°, 94.5% and 99.6% for GS 4° and 93.7% and 99.6% for GS 6°. The increase of the number of pixels failing the gamma criterion can be explained by the worse modelling of the continuous movement using a coarser gantry resolution. This model, the small-arc approximation, has theoretically been described by Webb and McQuaid [42]. We conclude that the approximation is still valid for GS 4°, when we compare the passing rates with IMRT, which are nearly the same. According to Feygelman et al. [32] and also suggested by Bzdusek et al. [25] we would use the largest GS consistent with good dosimetric results (GS 4°) to minimize the calculation time. Furthermore in this group nearly as many plans complied with the DVO (26) as in the GS 2° group (27). Only 21 were found with GS 6°. Consequently GS 6° is not recommended for VMAT planning of prostate cancer.

The IMRT calculations lack of a similar systematic variation of parameters as done for VMAT and therefore detailed statistic intercomparisons would not be appropriate. As the main parameters and DVO were taken (and adapted) from an IMRT study [27] the results should be quite characteristic. At most one might therefore expect an advantage for the IMRT plans. On the contrary to [25] no modifications to the DVO were made to improve VMAT plans. Figure 1 and Table 2 show that all VMAT groups with collimator 45° give comparable or better results for target volumes and OAR. IMRT attains the lowest number of MU. That is a discrepancy to other prostate planning studies referring to RapidArc® [12-14], but for the most part it is due to the fact that the number of MU in the mentioned studies with sliding window IMRT technique is higher than ours with step-and-shoot. This has also been observed by Alvarez-Moret et al. [18]. They also report that for equal or slightly more MU even a DA technique takes only 30% of an IMRT delivery time, for SA it is only 15%. Dobler et al. [19] list one prostate case with a time reduction from a seven field IMRT to a SA VMAT plan to 43% at comparable MU. Table 3 shows that our results for a larger number of patients confirm this benefit with statistical significance with a time reduction to around 50% for DA and to about 30% respectively 20% for SA.

It might be a surprise that higher MU are not generally related to improved plan quality. Obviously additional MU are not always exploited in smaller MLC apertures for better dose modulation. A similar effect has not only been observed in an IMRT TPS intercomparison [43], IMRT planning study comparisons of different algorithms within the same TPS [27,28,44], but also in the VMAT comparison of Palma et al. [14]. Surely there remains some potential to improve the algorithm.

Up to now the TPS does not offer the option of dynamic collimator rotation, which is technically available on the Elekta Synergy®S linear accelerator. As Webb has shown [45] this would help to avoid “parked gaps” for closed leaf pairs, which are needed during the treatment, but cannot be parked below the fixed diaphragm due to limited leaf speed. Avoiding such ‘unwanted fluence’ might be a next step to improve dose distributions in VMAT plans.

The results of this planning study may in detail be valid only for the chosen set of DVO. However the references [16,32] have shown that authors using different equipment and protocols but similar algorithms have achieved comparable results regarding GS and collimator angle. Although the majority of the plans failed the DVO, it could be shown that all planning aims are met using an appropriate set of parameters. The DVO for the rectum and for the PTV are somehow counterworking goals which are not met for all settings, as also was found by Crijns et al. [11], where all five RapidArc planning approaches failed achieving the rectum maximum dose.

## Conclusion

With the implementation of VMAT in the Oncentra® MasterPlan system many parameters must be kept in mind. For prostate planning there is a clear outcome that a collimator angle of 45° is advantageous. The plan quality and dosimetric results for GS 2° and 4° are comparable, but GS 4° reduces calculation time. SA treatments allow the fastest delivery in less than 2.5 min which is advantageous with respect to intrafractional organ movement, gaining a short delivery time and the best dose distribution with MDT 110 s. DA offers lower doses to the rectum with delivery times between 3 min and 5 min. Here MDT 80 s gives the best results.

## Abbreviations

CTV, Clinical target volume; DA, Dual arc; DVO, Dose volume objectives; GS, Gantry spacing; GTV, Gross target volume; IMRT, Intensity modulated radiation therapy; MDT, Maximum delivery time; MLC, Multi leaf collimator; MU, Monitor units; OAR, Organs at risk; OL, Patient outline; PTV, Planning target volume; PTVm, PTV plus margin; ROI, Regions of interest; R-PTVm, Rectum volume minus PTVm; SA, Single arc; SIB, Simultaneous integrated boost; TPS, Treatment planning system; VMAT, Volumetric modulated arc therapy.

## Competing interests

This work was partly supported by Theranostic GmbH, Solingen, Germany, the German branch of Nucletron B.V. (Veenendaal, Netherlands).

## Authors’ contributions

MT participated in the design of the study, performed planning and the statistical analysis, and drafted the manuscript, MH selected the patients, marked the ROI, and contributed to the discussion section. OK participated in the design of the study, BD participated in the concept and helped to draft the manuscript. All authors read and approved the final manuscript.
